# Identification of Antioxidant Proteins With Deep Learning From Sequence Information

**DOI:** 10.3389/fphar.2018.01036

**Published:** 2018-09-20

**Authors:** Lifen Shao, Hui Gao, Zhen Liu, Juan Feng, Lixia Tang, Hao Lin

**Affiliations:** ^1^Center for Informational Biology, School of Computer Science and Engineering, University of Electronic Science and Technology of China, Chengdu, China; ^2^Key Laboratory for Neuro-Information of Ministry of Education, Center for Informational Biology, School of Life Science and Technology, University of Electronic Science and Technology of China, Chengdu, China

**Keywords:** antioxidant proteins, deep learning, g-gap dipeptide, feature selection, webserver

## Abstract

Antioxidant proteins have been found closely linked to disease control for its ability to eliminate excess free radicals. Because of its medicinal value, the study of identifying antioxidant proteins is on the upsurge. Many machine-learning classifiers have performed poorly owing to the nonlinear and unbalanced nature of biological data. Recently, deep learning techniques showed advantages over many state-of-the-art machine learning methods in various fields. In this study, a deep learning based classifier was proposed to identify antioxidant proteins based on mixed g-gap dipeptide composition feature vector. The classifier employed deep autoencoder to extract nonlinear representation from raw input. The t-Distributed Stochastic Neighbor Embedding (t-SNE) was used for dimensionality reduction. Support vector machine was finally performed for classification. The classifier achieved *F*_1_ score of 0.8842 and *MCC* of 0.7409 in 10-fold cross validation. Experimental results show that our proposed method outperformed the traditional machine learning methods and could be a promising tool for antioxidant protein identification. For the convenience of others' scientific research, we have developed a user-friendly web server called IDAod for antioxidant protein identification, which can be accessed freely at http://bigroup.uestc.edu.cn/IDAod/.

## Introduction

Free radicals are a series of atoms, molecules or ions containing unpaired electrons, including reactive oxygen species (ROS such as hydroxyl radicals, superoxide anion and hydrogen peroxide) as well as reactive nitrogen species (RNS such as NO). Both ROS and RNS can be produced in cells via non-enzymatic reaction (e.g., Fenton reaction) or enzymatic catalytic reaction requiring NADPH oxidase, xanthine oxidase (XOD) or induced NO synthase etc. Appropriate amount of free radicals is essential for performing some physiological functions such as respiratory burst and liver detoxication. However, once the organisms suffer from environmental stresses, the level of ROS or RNS will be increased significantly in cells. The presence of excess free radicals will not only result in oxidative damage to proteins, DNA/RNA and the polyunsaturated fatty acids, but also regulate the activity of some protein kinases or transcription factors. Finally, it may cause changes in the expression of some genes and induce diseases such as diabetes, atherosclerosis, arthritis, cancer, and aging (Urso and Clarkson, 2003; Lee et al., 2004; Li et al., 2014). Obviously, the elimination of free radicals will greatly favor the treatment of these diseases.

It is generally known that the free radicals can be scavenged by various antioxidants (e.g., ascorbic acids, carotenoid, glutathione) and the antioxidant enzymes (such as Superoxide dismutase SOD, Catalases CAT, Peroxidase POD, and Glutathione peroxidase) within the cells. Numerous evidences have showed that different antioxidant proteins have different antioxidant mechanisms. For example, peroxiredoxins are one family member of thiol-containing POD which can remove H_2_O_2_ through the oxidation of cysteine residues to form -S-S bonds (Staudacher et al., 2018). SOD is a kind of Cu^2+^ or Mn^2+^-coordinated metalloenzyme, and it can effectively eliminate the harmful superoxide anion (Case, 2017). Different from SOD, CAT utilizes the cofactor (iron heme) to convert H_2_O_2_ to H_2_O and O_2_ (Alfonso-Prieto et al., 2009). These complexity and diversities make it time-consuming to identify antioxidant proteins through biochemical experiments. There is therefore the urgent need to draw support from computational methods.

A few studies have been done to identify antioxidant proteins automatically. Feng et al. adopted Naïve Bayes to predict antioxidant proteins (Feng et al., 2013) which achieved a performance accuracy up to 66.88% and sensitivity of 72.04% in jackknife test. Subsequently, they proposed a support vector machine (SVM) classification model based on dipeptide composition to predict antioxidant proteins (Feng et al., 2016). This model achieved higher accuracy of 74.79% in the jackknife test but its result in 10-fold cross validation showed poor ability to identify antioxidants. Besides, its feature selection method ANOVA may be oversimplified and the model was trained on single g-gap feature. Afterward an ensemble model was adopted based on secondary structure information, mutation probability and solvent accessibility (Zhang et al., 2016). The model achieved accuracy of 86.3% and sensitivity of 87.8% on independent testing dataset. However, all extracted features were completely dependent on software developed by others, leading to restricted versatility of the model. In addition, the features they used are more complicated including secondary structure information and amino acid mutations. In conclusion, there is still a long way to go in developing reliable and effective computational methods for antioxidant proteins identification.

Recently, deep learning makes a hit because of its extraordinary performance on the field of image processing. Compared with traditional machine learning methods that rely on feature engineering, deep learning is proved to have advantages of automatically discovering representations needed for classification from raw data (LeCun et al., 2015). In bioinformatics, deep learning also has been successfully applied to predict protein structure, gene prediction and protein function (Spencer et al., 2015; Zhang S. W. et al., 2017; Zou et al., 2017; Wei et al., 2018).

Thus, this study raised a deep learning method to identify antioxidant proteins based on feature extraction method called g-gap dipeptide composition. The whole model was built in four steps: (1) Extract a mixed g-gap feature vector from the sequence information of each sample. (2) Build a deep autoencoder and full connect (FC) neural network on the g-gap feature vector to learn its compact representation. (3) Reduce the dimension of compact representation through t-SNE. (4) Identify antioxidant proteins using SVM classifier. To evaluate the performance of model, 10-fold cross validation was performed. Furthermore, we established a free online server called IDAod based on the proposed method to provide convenient service for scholars.

## Materials

Raw dataset was collected from UniProt database (release 2014_02). In consideration of redundant sequences, the following process was implemented to improve quality of datasets: (1) Retain sequences confirmed to obtain antioxidant activity in biological experiment. (2) Eliminate sequences containing nonstandard letters except 20 familiar amino acid alphabets for the reason of ambiguous meanings. After processing, 710 antioxidant protein sequences were regarded as positive samples and remaining 1,567 protein sequences as negative samples.

Furthermore, studies have shown that redundant samples will lead to unreliable training results (Chou, 2011). To avoid homology bias and redundancy, the CD-HIT program (Fu et al., 2012) was used to remove sequences that were more than 60% similarity to any sequence in positive and negative samples. Furthermore, proteins containing nonstandard letters, like “B,” “X,” or “Z,” were excluded for their ambiguous meaning. Finally, the dataset contains 250 antioxidant protein sequences (positive samples) and 1,551 non-antioxidant protein sequences (negative samples).

## Methods

### Feature extraction

The function of protein is mainly decided by structure, amino acid composition of the sequence and the orders of residues (Hensen et al., 2012). Protein has the secondary structure and tertiary structure because of hydrogen bonding, hydrophobic bond, Van der Wasls forces and so on (Berg et al., 2002; Chen et al., 2014). The biological activity and physicochemical properties of proteins are mainly determined by the integrity of the spatial structure (Kim et al., 2003). Though protein primary sequence can't directly represent complete information, the researches (Zhu et al., 2015; Chen et al., 2016; Tang et al., 2016; Yang et al., 2016; Lai et al., 2017) on protein structure and function prediction using the information from primary sequence of protein indicate that protein primary sequence contains adequate information to predict the biological, physical and chemical properties of protein molecules. Thus, the feature described protein samples were also derived from protein primary sequence.

A protein with L amino acids can be formulated by *R*_1_*R*_2_*R*_3_*R*_4_…*R*_*L*−2_*R*_*L*−1_*R*_*L*_, where each *R*_*i*_ represent the ith residue of the protein. To extract the information from protein primary sequence as more as possible, we adopt g-gap dipeptide composition to transform a primary sequence to a vector. In secondary and tertiary structure, two non-adjoining residues are maybe connected by hydrogen bond. Thus, g-gap dipeptides compositions can reflect information about not only adjacent amino acids in sequence but also adjacent amino acids in space because of the hydrogen bonds. In detail, g is an integer ranging from 0 to 9. Each g-gap dipeptide feature vector *P*^*g*^ contains 20 × 20 = 400 dimensions and can be formulated as:

(1) $$\begin{array}{r} P^{g}={\left[f_{1}^{g}f_{2}^{g}\cdots f_{i}^{g}\cdots f_{400}^{g}\right]}^{T} \end{array}$$

where each element $f_{i}^{g}$ represent the frequency of the ith g-gap dipeptide in the protein sequence and is defined as:

(2) $$\begin{array}{r} f_{i}^{g}=\frac{n_{i}^{g}}{\sum_{i=1}^{400}n_{i}^{g}}=\frac{n_{i}^{g}}{\left(L-g-1\right)} \end{array}$$

where $n_{i}^{g}$ is the number of ith g-gap dipeptide in sequence. Let *A*_1_, *A*_2_, …, *A*_20_ represent 20 different kinds of amino acid, then 0-gap and 1-gap dipeptides can be expressed as *A*_*i*_*A*_*j*_ and *A*_i_*A_*j*_ respectively, where “^*^” represents any kind of amino acid. 0-gap dipeptide describes the correlation of two proximate residues. Generally, g-gap dipeptide indicates the correlation between two residues with the interval of g residues. In this study, only 0-gap dipeptide features and 1-gap dipeptide features are employed to form the input feature vector *P* which has 800 (400+400) dimensions for our model.

### Performance evaluation

To obtain reliable and stable model, cross validation (CV) is used to measure performance of models by splitting dataset into training set and validation set (Lin, 2008). Training set is used to build up model and validation set is used to measure properties of the model. Cross-validation is generally divided into three categories: 10-fold CV, jackknife CV and independent data test. 10-fold CV is finally put into use for the reason that jackknife CV is time consuming, and independent data is difficult to collect. In 10-fold CV, the whole dataset was equally split into 10 parts, and one part was chosen as the validation dataset while the remaining 9 parts was used as the training set to build the model.

For binary classification problem, the sensitivity (*S*_*n*_), specificity (*S*_*p*_), and accuracy (*Acc*) are often used to measure performance of classifier. *S*_*n*_ (*S*_*p*_) is also called recall of positive(negative) class.

(3) $$\begin{array}{r} \left\{ \begin{array}{c} S_{n}=\frac{TP}{TP+FN}\;\\ S_{p}=\frac{TN}{TN+FP}\;\\ A_{cc}=\frac{TP+TN}{TP+FN+TN+FP}\; \end{array}\right. \end{array}$$

where *TP, FP, TN*, and *FN* represent true positive, false positive, true negative, and false negative of all samples, respectively.

Usually, *S*_*p*_ and *S*_*n*_ tend to restrain each other, high *S*_*n*_ leads to low *S*_*p*_ and vice versa. Thus another index, *F*_1_ score, is the harmonic mean of precision and sensitivity, to measure the quality of a model. *F*_1_ score is formulated as:

(4) $$\begin{array}{r} F_{1}score=\frac{2\times precision\times Sn}{precision+Sn} \end{array}$$

where precision is defined as:

(5) $$\begin{array}{r} Precision=\frac{TP}{TP+FP} \end{array}$$

Matthews correlation coefficient (*MCC*) is another index to describe performance of machine learning model defined as following:

(6) $$\begin{array}{r} MCC=\frac{TP\times TN-FP\times FN}{\sqrt{\left(TP+FN\right)\left(TP+FP\right)\left(TN+FP\right)\left(TN+FN\right)}} \end{array}$$

In this study, antioxidant protein is referred as positive class and no-antioxidant protein as negative class.

### Feature selection

**Figure 1:**
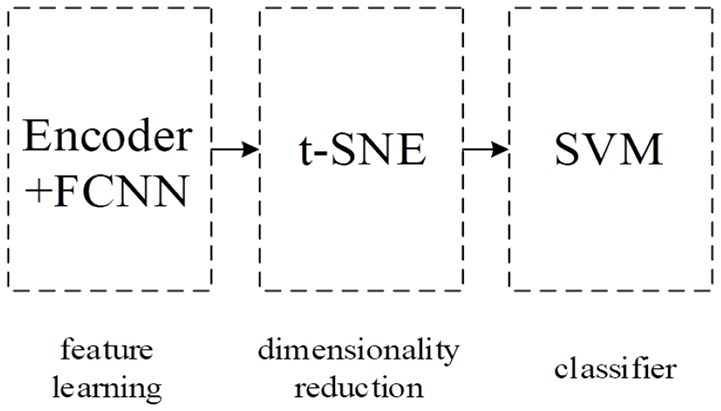
Our integral model's pipeline.

Our integral model IDAod is composed of three parts: encoder and FC layers part to learn the compressed representation of the g-gap feature vector, t-Distributed Stochastic Neighbor Embedding (t-SNE) part for reducing data dimensions (Maaten and Hinton, 2008) and SVM classifier part for the final identification of antioxidant proteins. Figure 1 shows the pipeline of our integral model.

#### Feature learning

**Figure 2:**
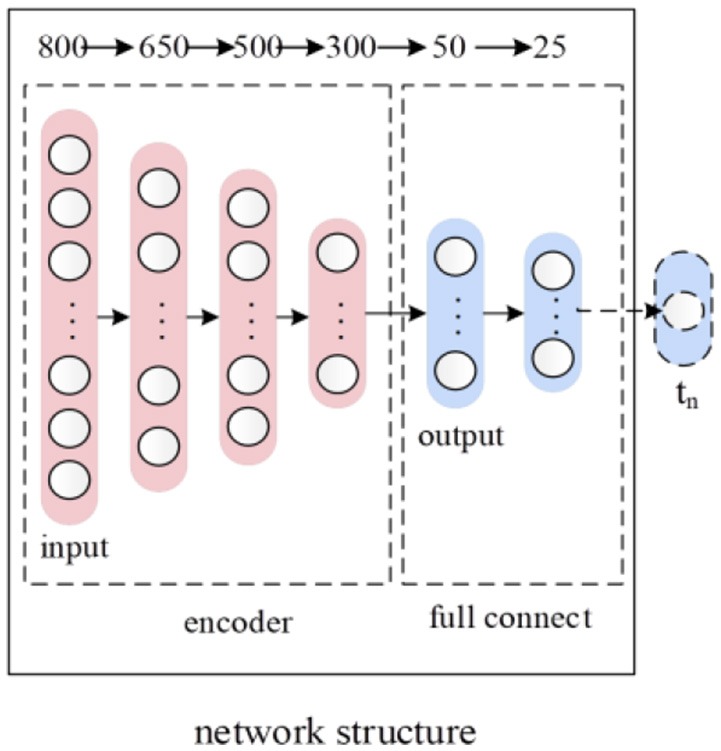
The deep neural network structure corresponding to feature learning.

Feature reduction and learning are effective for precision improvement. They have been applied successfully in many bioinformatics problems (Zou et al., 2016; Tang et al., 2017; Wei et al., 2017). For the feature learning, we first built an autoencoder (AE) (Vincent et al., 2010) that the input layer followed by an encoder and decoder then connected to the output layer. For reconstructing its original inputs, the output layer has the same number of nodes as the input layer. We then pre-trained the autoencoder to learn a dense representation of the input feature vector *P* that we obtained in section Feature Extraction. After the pre-training, we removed the decoder part of the autoencoder, and stack three layers of FC neural network on top of the encoder part of the autoencoder. After building the model, the deep neural network is fine-tuned, and the class label of the data servers as the target value. The deep neural network structure corresponding to feature learning was shown in Figure 2. The numbers at the top of Figure 2 indicate the number of nodes in each layer. The red After the feature learning, the output of the first FC layer was the final low-dimensional feature representation of the 800 dimensions of the input feature vector *P*.

#### Autoencoder(AE)

AE is an unsupervised dimensionality reduction method. It has been widely used to discover more abstract features of the raw data, which is specifically beneficial to the performance of the prediction and classification. A classical autoencoder is comprised of three layers: input layer, encoder layer and decoder layer. Encoder layer performs compression operation, and decoder layer performs uncompressing operation based on the output of encoder layer to reconstruct the original input.

**Figure 3:**
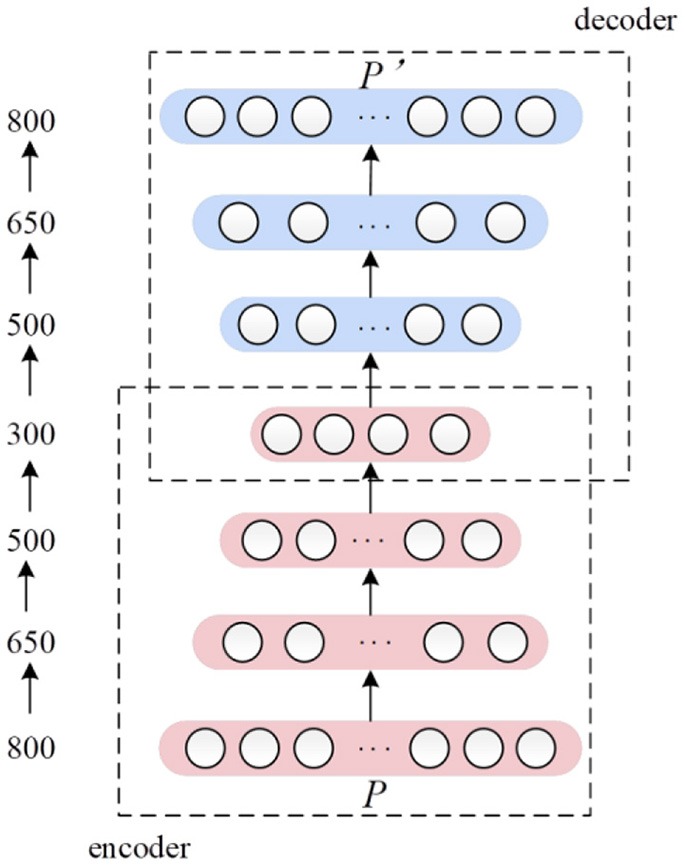
The architecture of the seven-layers' deep autoencoder. The number on the left side indicates the number of nodes in each layer.

Deep autoencoder is a kind of transformation of traditional AE. AE can only pre-train one encoder and decoder after each training, therefore we stacked multiple encode layers and corresponding decoder layers together to form a deep AE network. Afterwards the deep AE was trained jointly, with all the parameters optimized to minimize global reconstruction error between input and final decoder layer output. The architecture of our deep AE was showed as Figure 3.

In this paper, the deep AE consisted of 7 layers. The input layer with 800 nodes corresponded to 800 dimensions of the input feature vector *P*. Nodes' number of three encoder layers were 650, 500, and 300, respectively. Three decoder layers have 500, 650, and 800 nodes correspondingly. In the encoder and decoder framework, rectified linear network (ReLu) function was used as activation function. As learning is far easier in deep rectified linear network than in deep networks, we used sigmoid as activation function (Glorot et al., 2011).

The goal of autoencoder was to minimize the discrepancy between input and the reconstruction, thus the loss function can be defined as reconstruction error of original input *P* to reconstruction *P*′:

(7) $$\begin{array}{r} L\left(P,\;P^{\prime }\right)=\;||P-P^{\prime }|{|}^{2} \end{array}$$

Adadelta optimizer was employed to tune parameter in model to minimize the loss function (Zeiler, 2012). It can be seen from the loss function that samples of a large number of categories have a greater impact on the loss function. In our case of training dataset, the number of negative samples was around 6 times that of positive samples. In order to ease the excess impact from negative samples, we reassigned sample weight to 6 for each positive sample and to 1 for every negative sample.

#### Full connect neural network

After layer-wise unsupervised pre-training of deep autoencoder, 3 encoder layers were initialized with appropriate weights. Our goal was to identify antioxidant proteins, and 3 layers of FC neural network were thus stacked on top of autoencoder network to play the role of prediction. Node numbers of 2 hidden FC layers are 50 and 25 respectively. The output layer was a logistic regression classifier which consists of one node. After building, the deep neural network model for feature learning was fine-tuned on complete datasets where the class label of the input feature vector *P* served as the target value. The parameters of the whole model for feature learning will be tuned to minimize the gap between target values and the predicted values, and binary cross entropy is employed as loss function. Among total N samples, let *t*_*i*_ and *y*_*i*_ represent target label and predicted label of the ith sample respectively. Thus the binary cross entropy of dataset was defined as below:

(8) $$\begin{array}{r} \text{L}\left(\text{t},\text{y}\right)=-\sum_{i=1}^{N}\left\{t_{i}\ln y_{i}+(1-t_{i})\text{ln}(1-y_{i})\right\} \end{array}$$

A tanh function was used as activation function for two FC hidden layers. We adopted mini-batch gradient descent optimizer (Li et al., 2014), a variant of Stochastic Gradient Descent (SGD), to adjust parameters with the settings (lr = 0.0025, decay = 1e-6, monument = 0.6, batch size = 12), where lr was the learning rate, decay was a parameter reducing learning rate over each update to decrease vibration phenomenon for oversize learning rate. Monument setting was a trick to speed up training process by increasing update scale of parameters. Batch size was number of training data included to compute gradient of loss function. In SGD, batch size was 1. Unlike autoencoder initialized by unsupervised pre-training, weights of each FC layers were initialized randomly from −0.05 to 0.05 and biases are initialized to 0.

To improve generalization performance, dropout technique (Srivastava et al., 2014) was put into use in 2 hidden FC layers which was set to 0.2 and 0.3, respectively. In addition, we applied max norm constraints on weights of FC layers, the constraints limited the length of weights to less than or equal to 3.

Just as we did in the pre-training of AE, in the fine-tuning process, we re-weighted the ratio of positive sample to negative sample from 1:1 to 6:1.

### t-distributed stochastic neighbor embedding

Based on neural network shown in Figure 2, we reduce dimension of its output to two using t-SNE. t-SNE is a technique for dimensionality reduction that is well suited for visualization of high-dimensional datasets. Thus we convert each protein sequence into a point in two-dimensional space. Lastly, SVM plays the role of a classifier.

**Figure 4:**
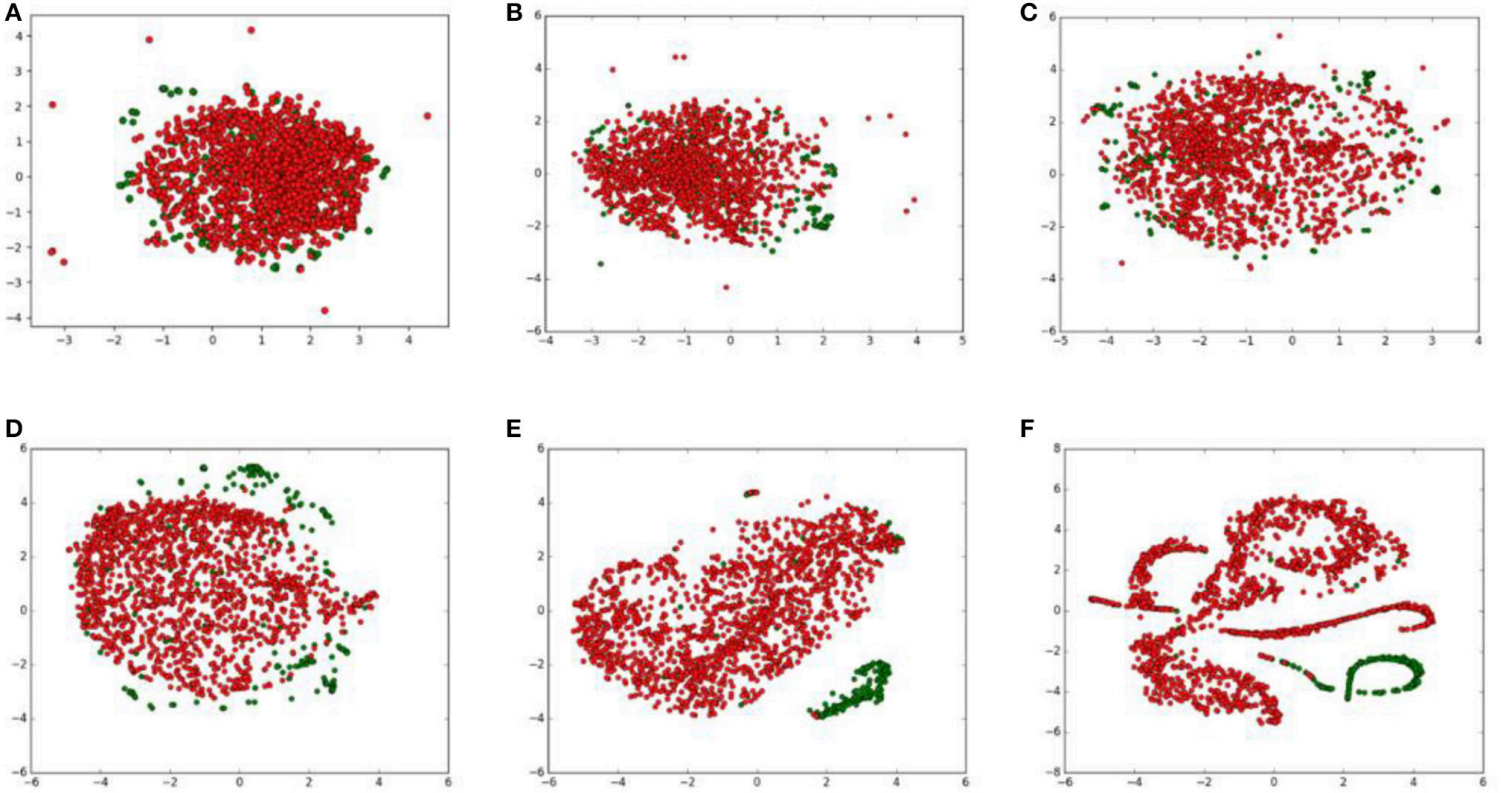
t-SNE visualization of the output of each layer in the deep neural network. Positive and negative samples are marked as green and red points, respectively. **(A)** input layer; **(B)** the first encoder layer; **(C)** the second encoder layer; **(D)** the third encoder layer; **(E)** the first FC layer; **(F)** the second FC layer.

To show how raw data will be transformed through each layer, we visualized outputs of hidden layers that have been reduced to 2 dimensions through t-SNE. The outputs of hidden layers were shown in Figure 4.

From the t-SNE transformation of hidden layers' outputs, we found that data points of two classes were mixed together after the first encoder layer. After transforming following encoder layers, data points of two classes gradually split. In the first FC layers, samples of two classes were almost separated obviously. The process shows autoencoder and FC neural network indeed extracts discriminate features and output of the first FC layers can be separated through t-SNE, thus the following classifier can play good performance based on those features.

### Support vector machine(SVM)

SVM is a powerful classifier for classification of linear and nonlinear classification problems (Adankon and Cheriet, 2009) and has been widely used in bioinformatics (Feng et al., 2013; Ding et al., 2014; Chen et al., 2016, 2018; Zhao et al., 2017; Su et al., 2018). Therefore, we used it to predict label on two-dimensional data transformed by t-SNE. Considering the small amount of data we have, linear kernel was used. Other parameters including penalty parameter C of the error term and class weight of two classes was tuned to reach the highest *F*_1_ score in 10-fold cross validation experiment. Finally, C was set to 2, and class weight of positive and negative class was set to 5:1. The SVM implement was based on method of freely available package scikit-learn (Pedregosa et al., 2012).

## Results and discussion

### Prediction accuracy

To evaluate the predictive performance of the final model, we compared our proposed model IDAod with AodPred (Feng et al., 2016), other frequently used classifiers like logistic, decision tree, random forest implemented in WEKA (Frank et al., 2004), and the ensemble model proposed by Zhang et al. (Zhang et al., 2016). In order to facilitate the comparison of experimental results, we reproduced AodPred in 10-fold cross validation. The 10-fold CV results of these classifiers based on 0-gap and 1-gap features of benchmark dataset are shown in Table 1. It's clearly reflected from the table that IDAod outperforms AodPred and other traditional machine learning methods with *S*_*n*_ of 81.27%, *S*_*p*_ of 99.59%, *F*_1_ score of 0.8842 and MCC of 0.7409. What's more is, IDAod based on simple g-gap dipeptide composition extracted directly from the primary sequence still outperforms the ensemble model that uses more complicated features including secondary structure information and amino acid site mutation information, etc.

**Table 1:** Comparison of our model with other methods.

**Methods**	**S_n_ (%)**	**S_p_ (%)**	**Acc (%)**	**F_1_ score**	**MCC**
AodPred	35.97	98.52	94.84	0.4959	0.4951
Logistic	53.23	80.38	76.39	0.3831	0.2695
Decision Tree	52.69	71.79	68.78	0.3230	0.1817
Random Forest	30.09	92.96	84.33	0.3465	0.2620
Ensemble Model	87.80	86.00	86.30	0.6699	0.6170
IDAod	81.27	99.59	97.05	0.8842	0.7409

Apart from the fact that the Ensemble Model attains the highest sensitivity (Sn) among all compared methods as is shown in Table 1, our IDAod method outperforms all other benchmark classifying methods on most performance indices, including Sp, Acc, F1 score, and MCC. There's usually a trade-off between sensitivity and specificity. In other words, to achieve high TPR (true positive rate), the sacrifice of TNR (true negative rate) is often needed, which means negative samples are more likely to be wrongly predicted to be positive, and thus more wet-lab experiments are needed in practice to perform the verification. Compared with using Ensemble Model, the sensitivity (Sn) using our method drops from 87.8 to 81.27% since our method performs more “rigorous” prediction for positive samples, and we pay more attention to the “average” score like accuracy, *F*_1_ score and MCC indicators for the sake of fairness. The results show IDAod outperforms the other classifiers. It reflects that IDAod can learn more abundant and discriminative features from mixed g-gap dipeptide composition. Therefore, IDAod can be a more advanced antioxidant protein identification too.

### Hyperparameters in network

The network architecture design is significantly important when adopting deep learning method. The selection of ANN types, layers, nodes, dropout, learning rate and so froth could be a tedious task. We performed a lot of experiments to find intuition on network designing, basically, using manual attempts. After the main architecture (autoencoder + FC + t-SNE) has been decided, we applied one of Bayesian optimization methods (Snoek et al., 2012) to determine the hyperparameters. Bayesian optimization is an automatic tuning approach for optimizing the performance of a given learning algorithm by modeling the algorithm's generalization performance through sampling the hyperparameters from a Gaussian process. For each model, a small subset of training data was used to train different models with different hyperparameters suggested iteratively by the Bayesian optimization. After enough iterations, the best performance will not improve and the optimal hyperparameters which get the best performance were used in the final model. By rigorous cross validation our model showed reliable and optimizing result in the last line of Table 1 named IDAod.

### Web server

Generally, user-friendly and publicly accessible web-servers (Lin et al., 2014, 2017; Tang et al., 2016, 2017; Yang et al., 2016; Chen et al., 2017; Li et al., 2017; Feng et al., 2018) or databases (He et al., 2015; Cui et al., 2017; Feng et al., 2017; Liang et al., 2017; Yi et al., 2017; Zhang T. et al., 2017) represent the future bioinformatics direction. Thus, for the convenience of fellow researchers, an online web server called IDAod is provided at http://bigroup.uestc.edu.cn/IDAod. The input of the web server is a set of protein sequences, which can either be uploaded as a single file or copied/pasted into the input textbox. Note that the input protein sequence should be in the FASTA format. The FASTA format sequence consists of a single initial line beginning with a greater-than symbol (“>”), followed by lines of amino acid sequence. After submitting the protein sequences and clicking the “Submit” button, the predicted results will be shown on a new webpage. Instruction for prediction is presented on a new webpage if users click the “About” button. Datasets used in our experiment can be downloaded through “Data” button.

### Conclusion and future work

In this study, a deep learning-based classifier was proposed based on mixed g-gap dipeptide composition to predict antioxidant protein. Compared with existing methods, the designed classifier can achieve automatic extraction of features from raw input, and the mixed g-gap dipeptide features build good foundation for deep learning to extract more discriminative features than single g-gap dipeptide features. Besides, t-SNE was adopted in the model to reduce dimensions, and thus ease common over-fitting problem in deep learning. In the final result comparison, *F*_1_ score was put into use since it has taken both precision and recall into account. Comparison with AodPred shows that our experimental results in a 10-fold cross validation increased 78.3% on *F*_1_ score. The deep learning model was built on simple protein primary sequence and yielded good performance, suggesting that it may become a practical tool in antioxidant protein identification.

## Author contributions

HG and HL conceived and designed the experiments. LS, HG, HL, and ZL analyzed the data; LS and ZL implemented the algorithm and created the web-server; LS, HG, HL, ZL, JF, and LT performed the analysis and wrote the paper. All authors read and approved the final manuscript.

### Conflict of interest statement

The authors declare that the research was conducted in the absence of any commercial or financial relationships that could be construed as a potential conflict of interest.

## References

[B1] Adankon M. Cheriet M. (2009). Support vector machine, in Encyclopedia of Bometrics, eds Li S. Z. Jain A. (Boston, MA: Springer). 10.1007/978-0-387-73003-5_299

[B2] Alfonso-Prieto M. Biarnés X. Vidossich P. Rovira C. (2009). The molecular mechanism of the catalase reaction. J. Am. Chem. Soc. 131, 11751–11761. 10.1021/ja9018572 19653683

[B3] Berg J. Tymoczko J. Stryer L. Stryer L. (2002). Biochemistry, 5th Edn. New York, NY: WH Freeman.

[B4] Case A. J. (2017). On the origin of superoxide dismutase: an evolutionary perspective of superoxide-mediated redox signaling. Antioxidants 6:82. 10.3390/antiox6040082 29084153 PMC5745492

[B5] Chen W. Feng P. Ding H. Lin H. Chou K. C. (2016). Using deformation energy to analyze nucleosome positioning in genomes. Genomics 107, 69–75. 10.1016/j.ygeno.2015.12.005 26724497

[B6] Chen W. Feng P. Yang H. Ding H. Lin H. Chou K. C. (2018). iRNA-3typeA: identifying 3-types of modification at RNA's adenosine sites. Mol. Ther. Nucleic Acids 11, 468–474. 10.1016/j.omtn.2018.03.012 29858081 PMC5992483

[B7] Chen W. Yang H. Feng P. Ding H. Lin H. (2017). iDNA4mC: identifying DNA N4-methylcytosine sites based on nucleotide chemical properties. Bioinformatics 33, 3518–3523. 10.1093/bioinformatics/btx479 28961687

[B8] Chen Y. C. Cheng C. S. Tjong S. C. Yin H. S. Sue S. C. (2014). Case study of hydrogen bonding in a hydrophobic cavity. J. Phys. Chem. B 118, 14602–14611. 10.1021/jp5097053 25412145

[B9] Chou K. C. (2011). Some remarks on protein attribute prediction and pseudo amino acid composition. J. Theor. Biol. 273, 236–247. 10.1016/j.jtbi.2010.12.024 21168420 PMC7125570

[B10] Cui T. Zhang L. Huang Y. Yi Y. Tan P. Zhao Y.. (2017). MNDR v2. 0: an updated resource of ncRNA–disease associations in mammals. Nucleic Acids Res. 46, D371–D374. 10.1093/nar/gkx1025 29106639 PMC5753235

[B11] Ding H. Deng E. Z. Yuan L. F. Liu L. Lin H. Chen W.. (2014). iCTX-Type: a sequence-based predictor for identifying the types of conotoxins in targeting ion channels. Biomed Res. Int. 2014:286419. 10.1155/2014/286419 24991545 PMC4058692

[B12] Feng P. Chen W. Lin H. (2016). Identifying antioxidant proteins by using optimal dipeptide compositions. Interdis. Sci. Comput. Life Sci. 8, 186–191. 10.1007/s12539-015-0124-9 26345449

[B13] Feng P. Ding H. Lin H. Chen W. (2017). AOD: the antioxidant protein database. Sci. Rep. 7:7449. 10.1038/s41598-017-08115-6 28784999 PMC5547145

[B14] Feng P. Yang H. Ding H. Lin H. Chen W. Chou K. C. (2018). iDNA6mA-PseKNC: identifying DNA N6-methyladenosine sites by incorporating nucleotide physicochemical properties into PseKNC. Genomics 10.1016/j.ygeno.2018.01.005. [Epub ahead of print]. 29360500

[B15] Feng P. M. Lin H. Chen W. (2013). Identification of antioxidants from sequence information using Naive Bayes. Comput. Math. Methods Med. 2013:567529. 10.1155/2013/567529 24062796 PMC3766563

[B16] Frank E. Hall M. Trigg L. Holmes G. Witten I. H. (2004). Data mining in bioinformatics using Weka. Bioinformatics 20, 2479–2481. 10.1093/bioinformatics/bth261 15073010

[B17] Fu L. Niu B. Zhu Z. Wu S. Li W. (2012). CD-HIT: accelerated for clustering the next-generation sequencing data. Bioinformatics 28, 3150–3152. 10.1093/bioinformatics/bts565 23060610 PMC3516142

[B18] Glorot X. Bordes A. Bengio Y. (2011). Deep sparse rectifier neural networks, in Proceedings of the 14th International Conference on Artificial Intelligence and Statistics (Fort Lauderdale, FL), 315–323.

[B19] He B. Chai G. Duan Y. Yan Z. Qiu L. Zhang H.. (2015). BDB: biopanning data bank. Nucleic Acids Res. 44, D1127–D1132. 10.1093/nar/gkv1100 26503249 PMC4702802

[B20] Hensen U. Meyer T. Haas J. Rex R. Vriend G. Grubmüller H. (2012). Exploring protein dynamics space: the dynasome as the missing link between protein structure and function. PLoS ONE 7:e33931. 10.1371/journal.pone.0033931 22606222 PMC3350514

[B21] Kim S. H. Shin D. H. Choi I. G. Schulze-Gahmen U. Chen S. Kim R. (2003). Structure-based functional inference in structural genomics. J. Struct. Funct. Genomics 4, 129–135. 10.1023/A:1026200610644 14649297

[B22] Lai H. Y. Chen X. X. Chen W. Tang H. Lin H. (2017). Sequence-based predictive modeling to identify cancerlectins. Oncotarget 8:28169. 10.18632/oncotarget.15963 28423655 PMC5438640

[B23] LeCun Y. Bengio Y. Hinton G. (2015). Deep learning. Nature 521:436. 10.1038/nature14539 26017442

[B24] Lee J. Koo N. Min D. B. (2004). Reactive oxygen species, aging, and antioxidative nutraceuticals. Compr. Rev. Food Sci. Food Safety 3, 21–33. 10.1111/j.1541-4337.2004.tb00058.x 33430557

[B25] Li M. Zhang T. Chen Y. Smola A. J. (2014). Efficient mini-batch training for stochastic optimization, in Proceedings of the 20th ACM SIGKDD International Conference on Knowledge Discovery and Data Mining (New York, NY: ACM), 661–670.

[B26] Li N. Kang J. Jiang L. He B. Lin H. Huang J. (2017). PSBinder: a web service for predicting polystyrene surface-binding peptides. Biomed Res. Int. 2017:5761517. 10.1155/2017/5761517 29445741 PMC5763211

[B27] Liang Z. Y. Lai H. Y. Yang H. Zhang C. J. Yang H. Wei H. H.. (2017). Pro54DB: a database for experimentally verified sigma-54 promoters. Bioinformatics 33, 467–469. 10.1093/bioinformatics/btw630 28171531

[B28] Lin H. (2008). The modified Mahalanobis Discriminant for predicting outer membrane proteins by using Chou's pseudo amino acid composition. J. Theor. Biol. 252, 350–356. 10.1016/j.jtbi.2008.02.004 18355838

[B29] Lin H. Deng E. Z. Ding H. Chen W. Chou K. C. (2014). iPro54-PseKNC: a sequence-based predictor for identifying sigma-54 promoters in prokaryote with pseudo k-tuple nucleotide composition. Nucleic Acids Res. 42, 12961–12972. 10.1093/nar/gku1019 25361964 PMC4245931

[B30] Lin H. Liang Z. Y. Tang H. Chen W. (2017). Identifying sigma70 promoters with novel pseudo nucleotide composition. IEEE/ACM Trans. Comput. Biol. Bioinform. 10.1109/TCBB.2017.2666141. [Epub ahead of print]. 28186907

[B31] Maaten L. V. D. Hinton G. (2008). Visualizing data using t-SNE. J. Mach. Learn. Res. 9, 2579–2605.

[B32] Pedregosa F. Gramfort A. Michel V. Thirion B. Grisel O. Blondel M. (2012). Scikit-learn: machine learning in python. J. Mach. Learn. Res. 12, 2825–2830.

[B33] Snoek J. Larochelle H. Adams R. P. (2012). Practical Bayesian optimization of machine learning algorithms, in International Conference on Neural Information Processing Systems (Guangzhou), 2951–2959.

[B34] Spencer M. Eickholt J. Jianlin Cheng (2015). A deep learning network approach to ab initio protein secondary structure prediction. IEEE/ACM Trans. Comput. Biol. Bioinform. 12, 103–112. 10.1109/TCBB.2014.2343960 25750595 PMC4348072

[B35] Srivastava N. Hinton G. Krizhevsky A. Sutskever I. Salakhutdinov R. (2014). Dropout: a simple way to prevent neural networks from overfitting. J. Mach. Learn. Res. 15, 1929–1958.

[B36] Staudacher V. Trujillo M. Diederichs T. Dick T. P. Radi R. Morgan B.. (2018). Redox-sensitive GFP fusions for monitoring the catalytic mechanism and inactivation of peroxiredoxins in living cells. Redox Biol. 14, 549–556. 10.1016/j.redox.2017.10.017 29128826 PMC5684490

[B37] Su Z. D. Huang Y. Zhang Z. Y. Zhao Y. W. Wang D. Chen W.. (2018). iLoc-lncRNA: predict the subcellular location of lncRNAs by incorporating octamer composition into general PseKNC. Bioinformatics 10.1093/bioinformatics/bty508. [Epub ahead of print]. 29931187

[B38] Tang H. Chen W. Lin H. (2016). Identification of immunoglobulins using Chou's pseudo amino acid composition with feature selection technique. Mol. Biosyst. 12, 1269–1275. 10.1039/C5MB00883B 26883492

[B39] Tang H. Zhang C. Chen R. Huang P. Duan C. Zou P. (2017). Identification of secretory proteins of malaria parasite by feature selection technique. Lett. Org. Chem. 14, 621–624. 10.2174/1570178614666170329155502

[B40] Urso M. L. Clarkson P. M. (2003). Oxidative stress, exercise, and antioxidant supplementation. Toxicology 189, 41–54. 10.1016/S0300-483X(03)00151-3 12821281

[B41] Vincent P. Larochelle H. Lajoie I. Bengio Y. Manzagol P. A. (2010). Stacked denoising autoencoders: learning useful representations in a deep network with a local denoising criterion. J. Mach. Learn. Res. 11, 3371–3408.

[B42] Wei L. Ding Y. Su R. Tang J. Zou Q. (2018). Prediction of human protein subcellular localization using deep learning. J. Paral. Distribut. Comput. 117, 212–217. 10.1016/j.jpdc.2017.08.009

[B43] Wei L. Xing P. Shi G. Ji Z. L. Zou Q. (2017). Fast prediction of protein methylation sites using a sequence-based feature selection technique. IEEE/ACM Trans. Comput. Biol. Bioinform. 10.1109/TCBB.2017.2670558. [Epub ahead of print]. 28222000

[B44] Yang H. Tang H. Chen X. X. Zhang C. J. Zhu P. P. Ding H.. (2016). Identification of secretory proteins in *Mycobacterium tuberculosis*using pseudo amino acid composition. Biomed Res. Int. 2016:5413903. 10.1155/2016/5413903 27597968 PMC4997101

[B45] Yi Y. Zhao Y. Li C. Zhang L. Huang H. Li Y.. (2017). RAID v2.0: an updated resource of RNA-associated interactions across organisms. Nucleic Acids Res. 45, D115–D118. 10.1093/nar/gkw1052 27899615 PMC5210540

[B46] Zeiler M. D. (2012). ADADELTA: an adaptive learning rate method. arXiv:1212.5701

[B47] Zhang L. Zhang C. Gao R. Yang R. Song Q. (2016). Sequence based prediction of antioxidant proteins using a classifier selection strategy. PLoS ONE 11:e0163274. 10.1371/journal.pone.0163274 27662651 PMC5035026

[B48] Zhang S. W. Jin X. Y. Zhang T. (2017). Gene prediction in metagenomic fragments with deep learning. Biomed Res. Int. 2017:4740354. 10.1155/2017/4740354 29250541 PMC5698827

[B49] Zhang T. Tan P. Wang L. Jin N. Li Y. Zhang L.. (2017). RNALocate: a resource for RNA subcellular localizations. Nucleic Acids Res. 45, D135–D138. 10.1093/nar/gkw728 27543076 PMC5210605

[B50] Zhao Y. W. Su Z. D. Yang W. Lin H. Chen W. Tang H. (2017). IonchanPred 2.0: a tool to predict ion channels and their types. Int. J. Mol. Sci. 18:1838. 10.3390/ijms18091838 28837067 PMC5618487

[B51] Zhu P. P. Li W. C. Zhong Z. J. Deng E. Z. Ding H. Chen W.. (2015). Predicting the subcellular localization of mycobacterial proteins by incorporating the optimal tripeptides into the general form of pseudo amino acid composition. Mol. Biosyst. 11, 558–563. 10.1039/C4MB00645C 25437899

[B52] Zou Q. Wan S. Ju Y. Tang J. Zeng X. (2016). Pretata: predicting TATA binding proteins with novel features and dimensionality reduction strategy. BMC Syst. Biol. 10:114. 10.1186/s12918-016-0353-5 28155714 PMC5259984

[B53] Zou X. Wang G. Yu G. (2017). Protein function prediction using deep restricted boltzmann machines. Biomed. Res. Int. 2017:1729301. 10.1155/2017/1729301 28744460 PMC5506480

